# Phosphodiesterase-5 inhibitors use and the risk of alzheimer's disease: a systematic review and meta-analysis

**DOI:** 10.1007/s10072-024-07583-9

**Published:** 2024-05-25

**Authors:** Moaz Elsayed Abouelmagd, Maickel Abdelmeseh, Amr Elrosasy, Yousef Hesham Saad, Asmaa Zakria Alnajjar, Mahmoud Eid, Atef Hassan, Abdallah Abbas

**Affiliations:** 1 Faculty of Medicine, Cairo University, Cairo, Egypt; 2 Faculty of Medicine, Alexandria University, Alexandria, Egypt; 3 Faculty of Medicine, Mansoura University, Mansoura, Egypt; 4 Faculty of Medicine, Al- Al-Azhar University, Gaza, Palestine; 5 Faculty of Medicine, October 6Th University, Cairo, Egypt; 6 Faculty of Medicine, Al-Azhar University, Damietta, Egypt

**Keywords:** Alzheimer's disease, Phosphodiesterase type 5 inhibitors, PDE5Is, Sildenafil, Dementia, Viagra

## Abstract

**Background:**

The management of Alzheimer's disease (AD) poses considerable challenges, necessitating the pursuit of innovative therapeutic approaches. Recent research has spotlighted the promising role of phosphodiesterase type 5 inhibitors (PDE5Is) in reducing the prevalence of AD, utilizing their vasodilatory properties to suggest a potential neuroprotective effect. This meta-analysis and systematic review aims to assess the relationship between the use of PDE5Is and the risk of AD.

**Methods:**

A detailed examination was carried out across several electronic databases till March 2024, including PubMed, Web of Science, Scopus, CENTRAL, and Embase. The focus was on identifying studies that compare the occurrence of AD among PDE5I users vs non-users. Through a random-effects model, pooled hazard ratios (HRs) were calculated, in alignment with guidelines from the Cochrane Handbook for Systematic Reviews and Meta-Analysis and the PRISMA standards.

**Results:**

This analysis included six studies, cumulating a participant count of 8,337,313, involving individuals treated with sildenafil, tadalafil, and vardenafil, against a control group undergoing other or no treatments. The cumulative HR for AD risk among PDE5I users versus the control group was 0.53 (95% CI: 0.32–0.86, p = 0.008), signaling a markedly reduced likelihood of AD development in the PDE5I group. Particularly, sildenafil usage showed a significant risk reduction (HR: 0.46, 95% CI: 0.31–0.70, p < 0.001), while findings for tadalafil and vardenafil were not significant. Test of subgroup differences found no difference between male and female participants in the risk of AD.

**Conclusions:**

Our findings suggest that the use of PDE5Is is associated with a reduced risk of AD, highlighting its potential as a protective agent against neurodegenerative diseases. Given the very low quality of evidence and the heterogeneity among the included studies, further high-quality research is warranted to confirm these findings and elucidate the underlying mechanisms. Register number PROSPERO 2024: CRD42024522197.

**Supplementary Information:**

The online version contains supplementary material available at 10.1007/s10072-024-07583-9.

## Introduction

Alzheimer's disease (AD) is a progressive neurodegenerative disorder that poses a significant public health burden. The prevalence of individuals aged 65 and above affected by Alzheimer's dementia in the United States is estimated to be approximately 6.7 million, and this figure is projected to rise to 13.8 million by the year 2060 [1]. AD currently ranks as the sixth-leading cause of death in the United States, with a notable increase of over 145% in AD-related deaths between 2000 and 2019 [2, 3]. Family caregivers play a substantial role in providing unpaid care, dedicating an estimated 18 billion hours of care in 2022 [4].

Alzheimer's disease is a complex condition influenced by a combination of genetic and environmental factors. Presently, there is no definitive treatment available for AD, and the existing therapeutic approach primarily focuses on managing the symptoms through the use of cholinesterase inhibitors, which serve as the first-line treatment option [5]. Consequently, there is a growing interest in the development of drugs that can potentially prevent the progression of AD, recognizing the need for interventions that target the underlying mechanisms of the disease [5].

Phosphodiesterase type 5 inhibitors (PDE5Is) are vasodilating drugs that block the degradative action of cGMP-specific PDE5 on cyclic GMP, leading to dilation of blood vessels and facilitating erection with sexual stimulation. They are used in the treatment of several diseases most commonly erectile dysfunction (ED) and pulmonary hypertension [6]. Commonly used FDA-approved PDE5Is include sildenafil, Tadalafil, Avanafil, and vardenafil [7]. These drugs have been found to have wider cardiovascular benefits and are being explored for the treatment of other conditions such as memory enhancement, cancer, and lower urinary tract symptoms [8].

PDE5 inhibitors have been investigated for their potential association with AD and related dementia. Preclinical investigations have indicated that PDE5Is can modulate the pathways associated with cyclic guanosine monophosphate, thereby enhancing neurovascular function, reducing neuroinflammation, and promoting neurogenesis [9]. Recent attention has been directed towards exploring the potential benefits of PDE5Is in AD. A recent trial focused on tadalafil, a specific PDE5I, aimed to evaluate its efficacy in cognitive impairment but yielded no statistically significant outcomes [10]. However, it is plausible that the beneficial effects of PDE5Is may be more apparent with long-term prophylactic usage rather than short-term administration in clinical trials. Therefore, longitudinal study designs may be more appropriate for investigating the potential benefits of PDE5Is.

Prior systematic reviews have underscored the significance of sildenafil and PDE5Is in both preventing and treating AD [9, 11, 12]. These reviews have identified substantial favorable effects of PDE5Is in animal studies related to AD. However, some reviews focused solely on animal trials [9], while others encompassed both animal and human trials [11, 12]. Notably, none of these reviews conducted a meta-analysis of longitudinal studies to assess the relationship between PDE5Is and the risk of AD. To the best of our knowledge, this systematic review and meta-analysis represent the first comprehensive attempt to evaluate the association between PDE5I usage and the risk of Alzheimer's disease.

## Methods

We conducted this study following the Cochrane Handbook of Systematic Review and Meta-analysis guidelines and the Preferred Reporting Items for Systematic Reviews and Meta-Analyses (PRISMA). The protocol was registered on the International Prospective Register of Systematic Reviews (PROSPERO); register number: CRD42024522197.

### Search strategy

We searched the following electronic databases for relevant keywords from conception until March 2024: PubMed, Web of Science, Scopus, CENTRAL, and Embase. For example, the search strategy for PubMed was:

("Phosphodiesterase Inhibitors" OR PDEI OR "avanafil" OR sildenafil OR tadalafil OR Viagra OR "vardenafil" OR "PDE5" OR "PDE5Is" OR "Erectile Dysfunction" OR "Premature Ejaculation" OR "Levitra" OR Cialis OR Stendra) AND ("ALZHEIMER'S disease" OR ALZHEIMER* OR dementia OR "major neurocognitive disorder" OR "cognitive decline" OR "cognitive impairment"). Additionally, Google Scholar and the bibliographies of included studies were used to search for potential studies.

### Study selection

After retrieving titles and abstracts from the previous step, we imported them into Rayyan [13], an online platform for screening studies in systematic reviews. Duplicates were removed, and 2 authors double-screened all records. M.E. resolved any conflicts in the selection process and conducted full-text screening of the eligible studies. Our inclusion and exclusion criteria were:

Inclusion criteria:

• English-language studies.
• Participants used at least one type of PDE5I for at least 1 year before AD diagnosis.
• Alzheimer’s disease was defined using validated diagnostic criteria, tools, or medical records.
• The studies included enough data for analysis.
• Longitudinal study design.
• A healthy control group free of PDE5I use.

Exclusion criteria:

• The full texts of the studies were unavailable after contacting the corresponding author.

### Data extraction and quality assessment

Two authors extracted relevant data from the included studies into a data extraction Google Sheet. They extracted the following data: study design, country, baseline characteristics, sociodemographic characteristics, measurement tools for AD, types of PDE5I used, number of participants, mean follow-up time, and key findings.

We assessed the quality of the studies using the Newcastle–Ottawa Quality Assessment Scale (NOS), which consists of eight questions and a maximum score of 9. The scale evaluates three parameters of quality: selection, comparability, and outcome/exposure [14]. To align with the standards set by the Agency for Healthcare Research and Quality, we applied the following thresholds to convert the NOS scores into ratings of good, fair, and poor quality to help [15]:

Good quality: 3 or 4 points in the selection domain, 1 or 2 points in the comparability domain, and 2 or 3 points in the outcome/exposure domain.

Fair quality: 2 points in the selection domain, 1 or 2 points in the comparability domain, and 2 or 3 points in the outcome/exposure domain.

Poor quality: 0 or 1 star in the selection domain, 0 points in the comparability domain, and 0 or 1 star in the outcome/exposure domain.

In addition, we utilized the GRADE approach to evaluate the overall quality of evidence and the strength of recommendations for outcomes. This approach considers factors such as risk of bias, inconsistency, indirectness, imprecision, and publication bias. The confidence in the estimates is categorized as high, moderate, low, or very low [16].

### Statistical analysis

The primary outcome of interest was the hazard ratio (HR) of AD in PDE5I-exposed individuals. The effect sizes of the included studies were expressed as hazard ratios (HRs) and one study as an odds ratio. If multiple outcomes in one study were reported, the outcomes with the best diagnostic criteria, the highest number of cases, and the best adjustment were chosen. An HR of 1 indicates no association, HR between 0 and 1 indicates a negative relationship, and HR greater than 1 indicates a positive relationship between PDE5I use and the risk of AD.

Heterogeneity between studies was assessed visually using forest plots and the I^2^ test. I^2^ values less than 50% indicated insignificant heterogeneity, while values ≥ 50% indicated significant heterogeneity. A fixed-effect model (inverse variance) or a random-effects model (DerSimonian‒Laird) was used to calculate the pooled HR and 95% confidence interval (CI) in cases of insignificant and significant heterogeneity, respectively. Sensitivity and subgroup analyses were performed to investigate potential sources of heterogeneity and to test the robustness of our results. We conducted subgroup analysis based on the type of PDE5I used and sex. Publication bias was not assessed due to the low number of studies.

## Results

### Search results and characteristics of the included studies

**Fig. 1:**
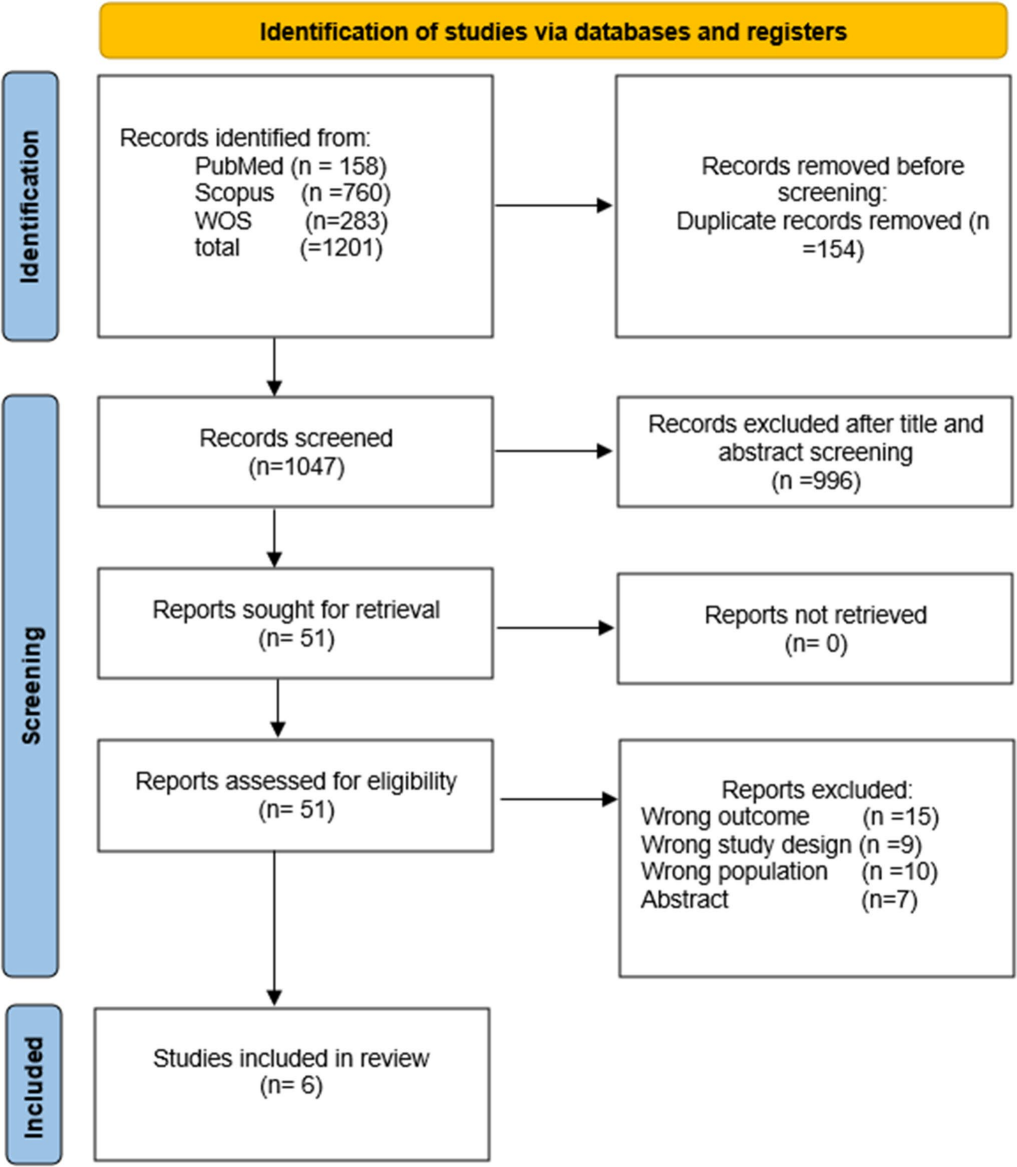
PRISMA flow diagram showing the details of screening and the study selection process

We retrieved 1201 papers through a literature search, 154 of which were duplicates, and we ultimately included six studies [17–22], presenting data from 8,337,313 patients in the MA (Fig. 1 and Table 1). We included three case–control studies [17–19], two prospective cohort studies [20, 21], and one retrospective cohort study [22]. The studies included data from the UK and the USA. The detailed characteristics of the included studies are shown in (Table 1).

**Table 1:** shows the characteristics and parameters of the included studies

Study ID	Country	Population (No. of events)	Types of PDE-5i	Male %	Age Mean (SD)	Summary of results	Total follow-up years (mean)	Quality of studies (NOS score)
Braun 2023 [20]	United States	5937 (248)	sildenafil, tadalafil AND vardenafil	100%	66 (9.8)	the use of PDE5i was not associated with dementia onset, and short-term medication use in younger men undergoing prostate cancer treatment may not increase the risk of Alzheimer's disease or related dementias	20 (11)	Poor Quality (5)
Adesuyan 2024 [21]	United Kingdom	269,725 (1,119)	sildenafil, tadalafil AND vardenafil	100%	58 (10)	PDE5I initiation in men with erectile dysfunction was associated with a lower risk of Alzheimer's disease, with a greater reduction in risk observed in individuals issued more than 20 prescriptions	16 (5.1)	Good quality (7)
Wilkinson2022 [17]	United Kingdom	551,344 (16,998)	sildenafil, tadalafil AND vardenafil	31%	74.52 (6.63)	Almost a third of medications were associated with dementia… Tadalafil was tentatively associated with lower dementia incidence	108 (17)	Fair Quality (6)
Huo 2023 [22]	United States	52,777(5,674)	Sildenafil	89%	NA	Sildenafil use was significantly associated with a 60% risk reduction of developing Alzheimer's disease (HR = 0.40; 95%CI:0.38–0.44; P < .0001) compared to the control group	3(-)	Good quality (6)
Henry 2023 [18]	United States	245,095 (17,565)	Not Specified	43.10%	75.57 (7.18)	Odds of PDE5i exposure were 64.2%, 55.7%, and 54.0% lower in patients with ADRD than controls among populations with erectile dysfunction, benign prostatic hyperplasia, and pulmonary hypertension, respectively	5 (-)	Good quality (8)
Fang 2023 [19]	United States	7,23 million (25,599)	Sildenafil	98%	74.2 (6.94)	sildenafil usage was significantly associated with a 69% reduced risk of AD (hazard ratio 0.31, 95% confidence interval 0.25–0.39, P < 1.0 × 10–8)."	6(-)	Poor Quality (5)

AD: Alzheimer’s disease; ADRD: Alzheimer’s disease and related dementia; PDE5I: phosphodiesterase-5 inhibitors; SD: standard deviation

### Quality assessment of the included studies

Four of the included studies were classified as having good or fair methodological quality [17, 18, 21, 22] (Fig. 1 and Table 1). These studies exhibited a strong adherence to methodological principles and minimal risk of bias, while the remaining studies were categorized as having poor quality [19, 20]. Using the GRADE system, all the met-analysis results yielded very low-quality evidence. Details and explanations are clarified in (Table 2).

**Table 2:** Summary of analyses and Quality of outcomes according to GRADE

Outcomes	Number of studies	Pooled HR (95% CI)	Heterogeneity (I2)	Test of subgroup difference (P value)	evidence (GRADE)
Primary Analysis					
Risk of AD	6	0.53 (0.32, 0.86)	99%		Very Low^b^
Gender				0.80	
Male	4	0.58 (0.31, 1.09)	99%		Very Low^b^
Female	2	0.53 ( 0.38, 0.73)	0%		Very Low^a,b^
Drug				1.00	
Sildenafil	4	0.46 (0.31, 0.70)	96%		Very Low^b^
Vardenafil	2	0.45 (0.12, 1.62)	96%		Very Low^a,b^
Tadalafil	2	0.44.(12, 1.64)	97%		Very Low^a,b^
Quality of studies				0.92	
Good or fair	4	0.54 (0.36, 80)	96%		-
Low	2	0.57 (0.17, 1.87)	99%		-

*HR: Hazard ratio; CI: Confidence interval; Explanations: a. Downgraded for a low number of studies included b. Downgraded for inconsistency due to high heterogeneity*

**GRADE Working Group grades of evidence:**

**High certainty:** we are very confident that the true effect lies close to that of the estimate of the effect

**Moderate certainty:** we are moderately confident in the effect estimate: the true effect is likely to be close to the estimate of the effect, but there is a possibility that it is substantially different

**Low certainty:** our confidence in the effect estimate is limited: the true effect may be substantially different from the estimate of the effect

**Very low certainty:** we have very little confidence in the effect estimate: the true effect is likely to be substantially different from the estimate of the effect

### Association between PDE5I use and risk of AD

**Fig. 2:**
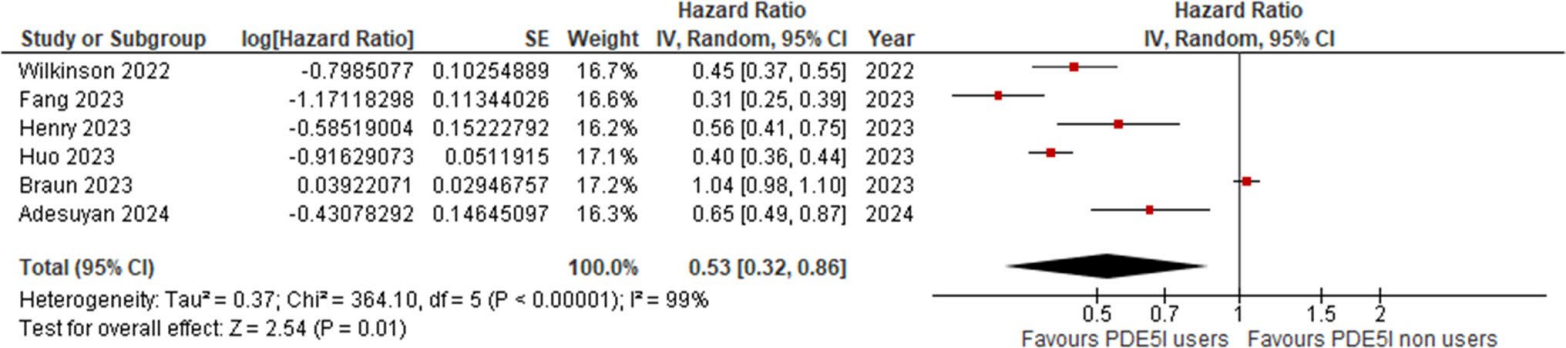
Forest plot of the risk of Alzheimer’s disease in PDE5Is users vs non-users

Six studies were included in the AD risk analysis [17–22]. All the studies assess the impact of PDE5I use on the risk of AD. The pooled HR was 0.53 [95% CI: 0.32, 0.86] indicating a statistically significant reduction in the risk of dementia AD for PDE5I users compared to non-users. Despite high heterogeneity (I^2 = 99%), the overall effect remains significant (p = 0.008). (Fig. 2).

#### Sensitivity analysis

We conducted a sensitivity analysis using a one-by-one exclusion method, removing one study at a time. After excluding Braun et al. [20], the heterogeneity was decreased to (I^2 = 81%) however still significant. Braun et al. study was conducted on prostatic cancer patients and had non-significant. The pooled analysis after exclusion resulted in HR of 0.45 [95% CI: 0.36, 0.55]. (Supplementary material Fig. 1).

### Risk of AD based on gender

**Fig. 3:**
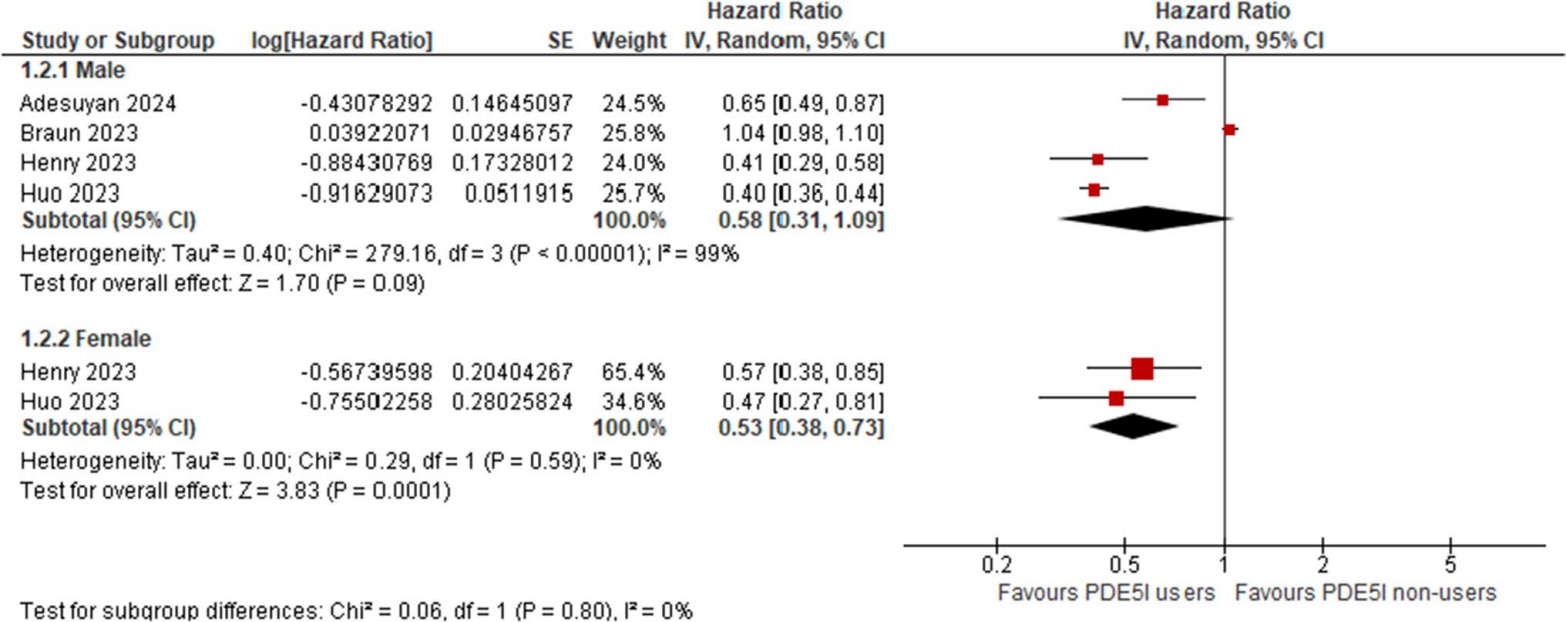
Forest plot of the subgroup analysis based on gender

Six studies were included in the analysis based on gender. Four studies were included in the male subgroup and two studies were included in the female subgroup. The male subgroup showed a non-significant decrease in the risk of AD with a pooled HR of 0.58 [95% CI: 0.31, 1.09] with significant heterogeneity (I^2 = 99%). However, the female subgroup showed a statistically significant decrease in AD risk with a pooled HR of 0.53 [95% CI: 0.38, 0.73] with no significant heterogeneity (I^2 = 0%) and the test for subgroup differences was not significant (P = 0.80). (Fig. 3).

### Risk of AD based on the drug used

Four studies were included in the analysis based on the drug used. Four studies were included in the sildenafil subgroup and two studies were included in the Vardenafil and Tadalafil subgroups. The sildenafil subgroup showed a statistically significant decrease in the risk of AD with an HR of 0.46 [95% CI: 0.31, 0.70; I^2 = 96%), but the Vardenafil and Tadalafil subgroups did not show a significant difference with an HR of 0.45 [95% CI: 0.12, 1.62; I^2 = 96%) and 0.44 [95% CI: 0.12, 1.64; I^2 = 97%] respectively. The subgroup differences test was insignificant (P = 1.00). (supplementary material Fig. 2).

## Discussion

Our meta-analysis investigated the potential impact of PDE5Is on the risk of AD. According to our findings, the use of PDE5Is significantly reduced the risk of AD in comparison to those who did not use it. Upon subgrouping the medications into sildenafil, vardenafil, and tadalafil, only sildenafil was found to reduce the risk of AD significantly. interestingly, subgrouping by sex showed significant effects for female but not male participants. However, these results are limited by the low number of studies.

Significant heterogeneity in the primary analysis limited the robustness of our studies. Some of the heterogeneity was resolved by the exclusion of Braun et al*.* [20]. The results of this study were not significant. While most studies investigated the use of PDE5Is in ED or benign urological conditions, this study was conducted among prostate cancer patients. The study included a low number of participants (5,937) men enrolled in the Cancer of the Prostatic Strategic Urologic Research Endeavor registry (CaPSURE) from 1998 to 2019. It is worth mentioning that the participants were not randomly selected and represented a younger and healthier cohort with lower incident AD rates compared to the general population. Consequently, there is a possibility that cases of AD might have been missed in this study.

Similarly, Adesuyan et al. [21], which had a young population, also reported a significant but weak negative association between the use of PDE5Is and the risk of AD. Adesuyan et al. [21] discovered that there was a reduced risk of AD in older adults who used sildenafil, but there was no significant evidence for a reduced risk with tadalafil or vardenafil. The association varied across different subgroups, with sildenafil showing a reduced risk in older men and individuals with a history of hypertension and diabetes.

Our overall findings may be affected by the dosage used and the duration of use, which requires further investigation. the current studies have some limitations that need to be considered when interpreting the results. For instance, all of the studies except Adesuyan et al*.* [21] did not investigate dose–response relationships due to the complexity of studying multiple drugs and the need for recorded data on the duration of PDE5I treatment. They recommended further studies to explore the role of single versus multiple prescriptions of PDE5Is and dose–response analyses. They conducted all analyses assuming individuals remained exposed after PDE5I initiation, regardless of subsequent discontinuation or treatment changes, similar to an intention-to-treat approach in randomized clinical trials.

The dose–response relationship between PDE5Is and their cognitive effects finds resonance in animal trials. In a recent systematic review of mice trials, two PDE5Is, icariin and icariside II, were found to have better cognitive effects with larger doses [9]. These drugs were associated with better resulting maze tests and decreased hippocampal deposition of β-amyloid -an important hallmark of AD. Notably, PDE5Is, particularly sildenafil, can cross the blood–brain barrier. Additionally, Sildenafil has been shown to suppress Aβ-42 and p-Tau, which play crucial roles in AD pathogenesis [23]. This suppression leads to the clearance of protein aggregates, shedding light on potential mechanisms underlying the effects of PDE5Is on AD risk.The observed reduction in AD risk aligned with previous research suggesting potential therapeutic effects of PDE5I. Fang et al*. *[19] found that sildenafil might have therapeutic effects in reducing the risk of AD. They evaluated the association between sildenafil usage and the risk of AD by analyzing data from large patient databases. Their findings showed that sildenafil usage was significantly associated with a reduced risk of AD, with a 69% reduction in risk. Even after adjusting for comorbidities like coronary artery disease, hypertension, and type 2 diabetes, sildenafil usage was still linked to a decreased risk of AD across various drug cohorts. These findings suggested that sildenafil might offer a potentially beneficial effect in reducing the risk of AD.

Henry et al*.* [18] noticed that patients with AD had a decreased likelihood of using PDE5Is compared to those without AD across all three FDA-approved indications for PDE5Is. This association was consistent in both males and females, with either sildenafil or tadalafil among patients with different comorbidities. Huo et al*.* [22] also found that sildenafil was significantly associated with a 62% lower risk of Alzheimer's disease in men and a 47% lower risk in women. It is worth mentioning that this study relied on retrospective data from the IBM MarketScan Database, which might have inherent biases and limitations in data accuracy. Additionally, the study did not account for potentially confounding variables such as lifestyle factors, genetic predisposition, or other medications that could influence the risk of Alzheimer's disease.

PDE5Is have established efficacy in the treatment of various conditions, such as erectile dysfunction (ED) and pulmonary hypertension [6]. However, considering the potential role of PDE5Is in preventing AD, it is important to consider the long-term side effects. Generally, PDE5Is are considered safe drugs, and commonly reported adverse events include headache, dizziness, and flushing [24]. Nonetheless, it is important to note that rare adverse events, such as hearing loss and priapism, may occur. Till now, the available evidence suggests that long-term use of PDE5Is is generally safe [25], but caution should be exercised, particularly of contraindications such as concomitant use with nitrates or alpha-blockers [26]. Our study is the first systematic review and meta-analysis aimed at exploring the risk of AD in users of PDE5Is compared to non-users. We included six studies with a substantial number of participants. Previous systematic reviews [11, 12] did not incorporate any longitudinal human studies or perform a meta-analysis of the available evidence. Additionally, we investigated the importance of the type of PDE5Is and the sex of participants. Although our initial findings suggest a potential association between PDE5I use and a decreased risk of AD, it is essential to exercise caution when interpreting these results due to the following limitations:

Firstly, the limited number of available studies restricted our ability to thoroughly investigate publication bias. Secondly, our analysis displayed high heterogeneity, which prompted us to conduct subgroup and sensitivity analyses. Although heterogeneity was not resolved, we deemed it appropriate to proceed with the meta-analysis considering the comparable designs of the included studies. Thirdly, the observational study designs employed in the included studies do not establish causal relationships but rather indicate associations. However, given the nature of our study, this design was the most practical and suitable approach. Fourth, the quality of evidence evaluated using the GRADE approach was determined to be very low for all outcomes. As the first meta-analysis on this topic, this underscores the importance of additional high-quality studies to strengthen the overall evidence base. Lastly, due to limited available data, we were unable to assess the dose–response relationship or the duration of PDE5I use.

Overall, while our study has notable limitations, it provides valuable insights into the current understanding of the association between PDE5I use and AD. As the world population continues to grow, the need for strategies to prevent AD development will increase [3]. PDE5Is are relatively safe, inexpensive, and widely accessible drugs [7] that can potentially play a role in these future prevention strategies.

Further research with larger sample sizes is necessary to enhance the robustness of the evidence in this field. Rigorous study designs and comprehensive assessment of dose–response relationships and treatment duration are also needed. It is important to investigate the relationship between PDE5I use and other types of dementia. Additionally, exploring the role of age, sex, and other covariates should be considered to determine the effectiveness of PDE5Is in reducing the risk of AD. By addressing these research gaps, we can advance our understanding of the potential benefits of PDE5Is in preventing AD and develop more targeted and effective approaches to combat this devastating neurodegenerative disease.

## Conclusion

In conclusion, our study suggests that the use of PDE5Is may play a significant role in the prevention of Alzheimer's disease. However, it is important to exercise caution when interpreting these results due to the limitations of our study. Further high-quality, longitudinal studies are necessary to address these limitations and provide more robust evidence. Understanding the potential benefits of PDE5Is in the prevention of AD is essential for gaining insights into the underlying disease mechanisms. It can also aid in the development of improved strategies to alleviate the global burden of Alzheimer's disease. By exploring the preventive effects of PDE5Is, we can potentially contribute to the development of AD risk reduction regimens to help clinicians make better-informed decisions for their patients and offer protection to individuals who may be at risk of developing AD.

## Supplementary Information

Below is the link to the electronic supplementary material.

Supplementary file1 (DOCX 42 KB)

## Data Availability

The data that support the findings of this study are available from the corresponding author upon reasonable request.

## Declarations

### Guarantor

Moaz Abouelmagd.
