# Supplementary material for: Phosphodiesterase-5 inhibitors use and the risk of alzheimer's disease: a systematic review and meta-analysis
Source: Neurol Sci. 2024 May 25;45(11):5261–70. doi: 10.1007/s10072-024-07583-9 (PMC11470851; doi:10.1007/s10072-024-07583-9)
Supplement: Supplementary file 1 — Supplementary file1 (DOCX 42 KB) [file 10072_2024_7583_MOESM1_ESM.docx]

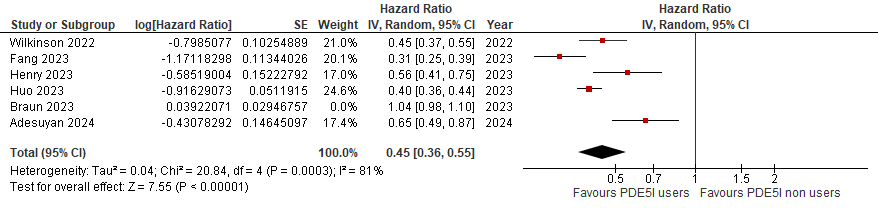


Fig.1 Sensitivity analysis of Association between PDE5I use and risk of dementia


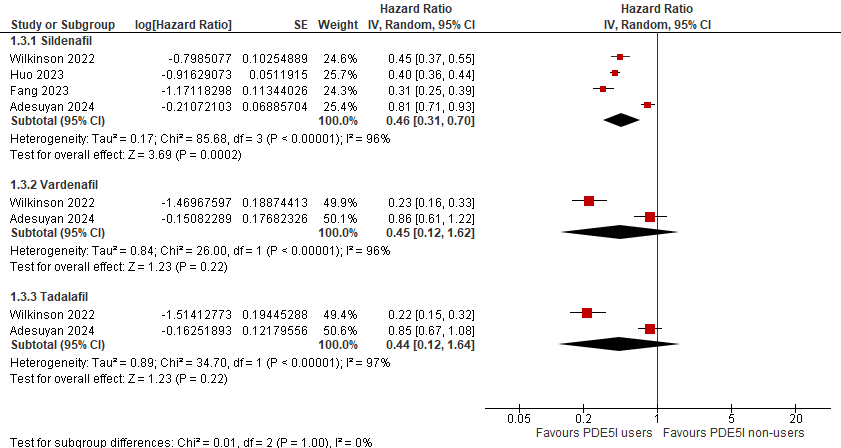


Fig.2 Subgroup analysis for the risk of dementia based on the drugs used
